# 

**Published:** 1841-10

**Authors:** 


### CONTENTS

OF NO. IV.

### ORIGINAL COMMUNICATIONS.

### ESSAYS AND CASES.

ART. I.—A Memoir on the Treatment of Bubo. By DR. MARCHAL (DE CALVI.) - - - - 241
ART. II.—History of a Case of Abdominal Disease. By WM. L. SUTTON, M. D. - - . - - 260
ART. III.—Observations on the Bloody Murrain of Cattle. By JOHN WINANS, M. D. - - - - 268

### REVIEWS.

ART. IV.—Statistical Researches relative to the Etiology of Pulmonary and Rheumatic Diseases, &c. By SAMUEL FORRY, M. D. - - - - - 271
Bibliographical Notice. - - - - - 294

### SELECTIONS FROM AMERICAN AND FOREIGN JOURNALS.

Case of Polypus Uteri, removed by Excision: with practical remarks. By CHANDLER R. GILMAN, M. D. - - 295
Excision of the Uvula and Tonsil, for the cure of Stammering. 299
Case of Calculous Concretion, &c. By WILLARD PARKER, M. D. - - - - - - 300
On the value of Albumen in the Urine, &c. By W. C. ROBERTS, M. D. - - - - - - 302

On Dilatation by Fluid Pressure in Stricture of the Urethra. By James Arnott, M. D. - - - - - 306
A Case of Ovarian Dropsy. By J. B. Kissam. - - 309
New Treatment of Hydrocele. By M. Jobert. - 313
Dislocation of the Thumb. - - - - - 313
Tetanus, cured by section of the nerve supplying the part. 314

ORIGINAL INTELLIGENCE.

Endemic Dysentery. - - - - - 315
Board of Health of New Orleans. - - - 315
State Medical Society of Kentucky. - - - 316
Coup de Soleil, or Stroke of the Sun. - - 317
Absence of Lacteal Secretion after Parturition. - - 317
Fistula in Ano. By M. L. Linton. - - 318
A Scientific Hoax. - - - - - 320
Medical Journals. - - - - - 320

